# Intelligent SERS Navigation System Guiding Brain Tumor Surgery by Intraoperatively Delineating the Metabolic Acidosis

**DOI:** 10.1002/advs.202104935

**Published:** 2022-01-12

**Authors:** Ziyi Jin, Qi Yue, Wenjia Duan, An Sui, Botao Zhao, Yinhui Deng, Yuting Zhai, Yuwen Zhang, Tao Sun, Guang‐Ping Zhang, Limei Han, Ying Mao, Jinhua Yu, Xiao‐Yong Zhang, Cong Li

**Affiliations:** ^1^ Key Laboratory of Smart Drug Delivery Ministry of Education State Key Laboratory of Medical Neurobiology School of Pharmacy Fudan University Shanghai 201203 China; ^2^ Department of neurosurgery Huashan Hospital Fudan University Shanghai 200040 China; ^3^ School of Information Science and Technology Fudan University Shanghai 200438 China; ^4^ Institute of Science and Technology for Brain‐Inspired Intelligence Fudan University Shanghai 200433 China; ^5^ MOE Key Laboratory of Computational Neuroscience and Brain‐Inspired Intelligence MOE Frontiers Center for Brain Science Shanghai China; ^6^ School of Physics and Electronics Shandong Normal University Jinan 250358 China

**Keywords:** artificial intelligence, brain tumor, metabolic acidosis, surface enhanced Raman spectroscopy, surgery navigation system

## Abstract

Surgeons face challenges in intraoperatively defining margin of brain tumors due to its infiltrative nature. Extracellular acidosis caused by metabolic reprogramming of cancer cells is a reliable marker for tumor infiltrative regions. Although the acidic margin‐guided surgery shows promise in improving surgical prognosis, its clinical transition is delayed by having the exogenous probes approved by the drug supervision authority. Here, an intelligent surface‐enhanced Raman scattering (SERS) navigation system delineating glioma acidic margins without administration of exogenous probes is reported. With assistance of this system, the metabolites at the tumor cutting edges can be nondestructively transferred within a water droplet to a SERS chip with pH sensitivity. Homemade deep learning model automatically processes the Raman spectra collected from the SERS chip and delineates the pH map of tumor resection bed with increased speed. Acidity correlated cancer cell density and proliferation level are demonstrated in tumor cutting edges of animal models and excised tissues from glioma patients. The overall survival of animal models post the SERS system guided surgery is significantly increased in comparison to the conventional strategy used in clinical practice. This SERS system holds the promise in accelerating clinical transition of acidic margin‐guided surgery for solid tumors with infiltrative nature.

## Graphical abstract

Surgeons face challenges in defining brain tumor margins due to its infiltrative nature. Here, an intelligent surface‐enhanced Raman scattering (SERS) navigation system is developed to intraoperatively guide tumor resection by delineating the metabolic acidosis without the administration of exogenous probes. Considering the correlation between tissue acidity and malignancy, this system holds the promise in improving surgical prognosis by precisely defining tumor infiltrative regions.

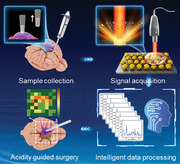

## 1 Introduction

Glioma accounts for more than 80% of all primary malignant brain tumors. Surgical removal is a mainstream treatment for glioma due to its benefits in debulking tumor volume, alleviating intracranial pressure and collecting tissue samples for biopsy purposes. However, because of the infiltrative nature of gliomas and textural similarities between malignant and normal brain tissues, neurosurgeons face the challenge of maximizing tumor resection while minimizing neurological deficits by preserving critical functional regions.^[^ ^1^, ^2^ ^]^ Therefore, it is essential to intraoperatively demarcate the infiltrated regions of glioma.

Although numerous techniques have been applied to identify glioma margins, few of them truly defines tumor infiltrative boundaries. For example, stereotactic navigation, the most widely used surgery guidance technique in clinical practice, allows the neurosurgeon to assess glioma location, volume, and boundaries by registering the anatomical landmarks in presurgical magnetic resonance images (MRI) with the tracked patient's rigid anatomy. However, these boundaries actually present the regions with a disrupted blood brain barrier (BBB) where the paramagnetic agent leaks out instead of the region with high malignancy.^[^ ^3^, ^4^ ^]^ Consequently, a strategy for intraoperatively locating the infiltrative regions is important to improve the surgical prognosis.

Metabolic reprogramming refers to the ability of cancer cells altering their metabolism to support their rapid proliferation.^[^ ^5^ ^]^ Metabolic reprogramming is generally accepted as a hallmark of cancer cells^[^ ^6^ ^]^ and provides them with survival advantages, such as establishment of the tumor microenvironment, stimulation of angiogenesis, and evasion of immune surveillance.^[^ ^7^, ^8^ ^]^ In comparison to structural disruption of the BBB, tumor associated metabolites are more reliable and earlier markers for malignant tissues.^[^ ^9^ ^]^ For example, choline derivatives visualized by MR spectroscopy, glycerophospholipids, and 2‐hydroxyglutarate detected by mass spectrometry (MS) have been used for delineating tumor margins^[^ ^10^, ^11^ ^]^ in clinical trials.^[^ ^12^ ^]^ However, the clinical translation of tumor metabolite‐guided surgery strategy faces challenges such as inadequate spatial resolution, time‐consuming acquisition, and inconvenient manipulation.^[^ ^13^ ^]^ Glucose metabolism shifting from oxidative phosphorylation to aerobic glycolysis, leading to acidification of the extracellular fluid (pH 6.2‐6.9), is an unequivocal hallmark of solid tumors.^[^ ^6^, ^14^ ^]^ Gillies et al. reported the spatiotemporal correlation between tissue acidity and malignancy in solid tumors.^[^ ^15^ ^]^ Gertler et al. showed that acidification at the tumor‐stroma interface drove transcriptome rewiring that promoted the acquisition of the most invasive phenotypes.^[^ ^16^ ^]^ Moreover, acidity promotes tumor progression by establishing an immunosuppressive environment^[^ ^7^, ^17^ ^]^ and driving glioma cells to a pluripotent, stem cell‐like state by upregulating stem cell markers.^[^ ^18^ ^]^ Intraoperatively visualizing and excising the acidic regions hold promise to improve surgical prognosis.

Surface‐enhanced Raman scattering (SERS) is a spectroscopic technique based on the plasmon‐assisted scattering of molecules absorbed on the noble metal surface.^[^ ^19^, ^20^ ^]^ In contrast to fluorescence imaging, SERS shows higher photostability, uncompromised sensitivity, and multiplexing potential for the simultaneous detection of up to 10 compounds.^[^ ^21^ ^]^ We previously reported pH‐responsive SERS nanoprobes that visualized acidic regions in glioma xenografts via acidity‐triggered Raman signal enhancement.^[^ ^22^ ^]^ Recently, we presented a ratiometrically pH‐responsive SERS probe delineating acidic margins by determining the pH values of tumor cutting edges.^[^ ^23^ ^]^ Even though these strategies markedly extended survival of animal models, their clinical transition is hindered by having the SERS nanoprobes approved by the drug supervision authority in a short term.^[^ ^19^, ^24^ ^]^ The translation of the acidic margin‐guided surgery will be accelerated if there is no need to inject the exogenous probes.

**Figure 1:**
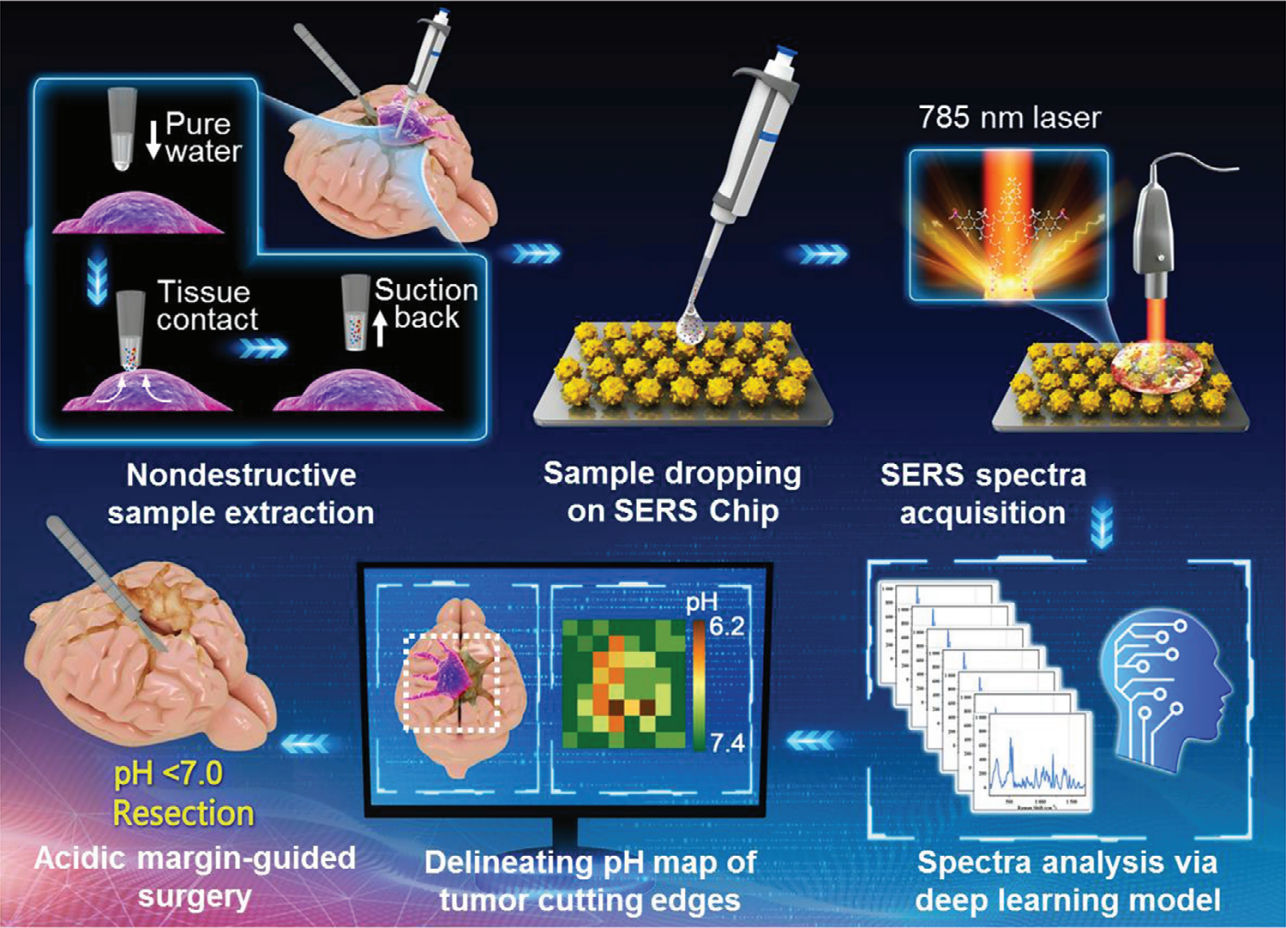
Schematic diagram of the surface‐enhanced Raman scattering (SERS) navigation system intraoperatively delineating acidic margin of glioma. A trace amount of pure water (≈0.4 µL) in the pipette tip contacts suspicious tissue at the tumor cutting edge for 2‒4 s. Then, the water droplet is sucked back and dripped onto a homemade pH‐sensitive SERS chip. The Raman spectra of the aqueous sample on the SERS chip were acquired by a handheld Raman scanner equipped with a 785 nm laser. The pH map of tumor cutting edges was intraoperatively delineated with the assistance of a deep learning model by automatically analyzing the Raman spectra. With the guidance of the pH map, acidic tissues with pH values less than 7.0 were excised.

In this work, we report a SERS navigation system for intraoperatively delineating the acidic margin of glioma without the administration of exogenous probe (**Figure** **1**). This system is composed of a SERS chip that senses sample's acidity via pH‐responsive Raman signals, a handheld Raman scanner that collects the Raman signal of the sample placed on the SERS chips, and a homemade deep learning model that determines sample's pH by automatically processing the Raman spectra. This SERS system demonstrates the acidity correlated cancer cell density and proliferation level in tumor cutting edges of rat models bearing orthotopic glioma allografts and excised tissues from glioma patients. Acidic margin‐guided surgery markedly increases overall survival compared to the models post the conventional surgical strategy used in clinical practice. Overall, this SERS navigation system not only confirms the feasibility of acidic margin‐guided surgery, but also provides a prototype to accelerate the clinical translation of this surgical strategy.

## 2 Results

### 2.1 Fabrication of the pH Responsive SERS Chip

**Figure 2:**
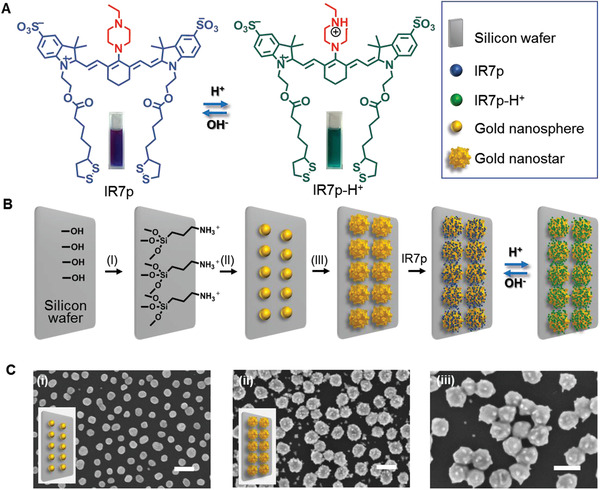
Fabrication of surface‐enhanced Raman scattering (SERS) chip with pH sensitivity. A) As a heptamethine cyanine derivative, the Raman reporter molecule IR7p showed a reversible color change between its protonated and deprotonated states. B) For developing the SERS chip, the silicon wafer surface was first functionalized with primary amines and then conjugated with gold nanospheres with a diameter of 45 nm via electrostatic affinity. The gold nanospheres further grew into nanostars with an average diameter of 70 nm. The nanostar surface was finally modified with IR7p. I) 2% (v/v) 3‐aminopropyl‐triethoxysilane in ethanol, 0.2 m HCl; II) gold nanospheres; and III) 0.3 × 10^−3^ m HAuCl_4_ in 75 × 10^−3^ m HEPES solution (pH 7.4). C) Scanning electron microscopy (SEM) images of gold nanospheres and nanostars on silicon wafer surfaces. i) Gold nanospheres, ii) gold nanostars, and iii) enlarged picture of nanostars. Scale bar: 100 nm.

The SERS chip was made up of SERS sensors fabricated on a silicon wafer (**Figure** **2**). The SERS sensors were comprised of gold nanostars and Raman reporter IR7p^[^ ^23^ ^]^ modified on nanostar surface. Notably, the reversible protonation and deprotonation of the piperazine group led to a remarkable color change in IR7p (Figure 2A). The SERS chip was prepared by three steps. First, the silicon wafer was cleaned thoroughly to expose the hydroxyl groups, which were converted to primary amines with positive charges. Second, the wafer surface was modified with negatively charged gold nanospheres^[^ ^23^ ^]^ via electrostatic affinity. Third, the gold nanospheres were grown into gold nanostars whose surface was further modified with IR7p via Au‐S bonds (Figure 2B). The brief synthesis of IR7p was shown in Figure S1 (Supporting Information). Scanning electron microscopy (SEM) studies showed that gold nanospheres and nanostars were both distributed homogeneously on the wafer surface. Compared to the nanospheres with an average diameter of 45 nm, the diameter of the gold nanostars increased to 70 nm (Figure 2C, S2, Supporting Information). Notably, each gold nanostar had 4–7 short branches with a length of 11 ± 4 nm. The proportion of nanoparticles in contact with at least one neighboring nanoparticle increased from 8.7% of the nanospheres to 85.3% of the nanostars. The extinction spectra of the nanospheres, nanostars, and nanostars coated with IR7p are shown in Figure S3 (Supporting Information).

### 2.2 Proposed Mechanisms Leading to the pH Sensitivity of the SERS Chip

**Figure 3:**
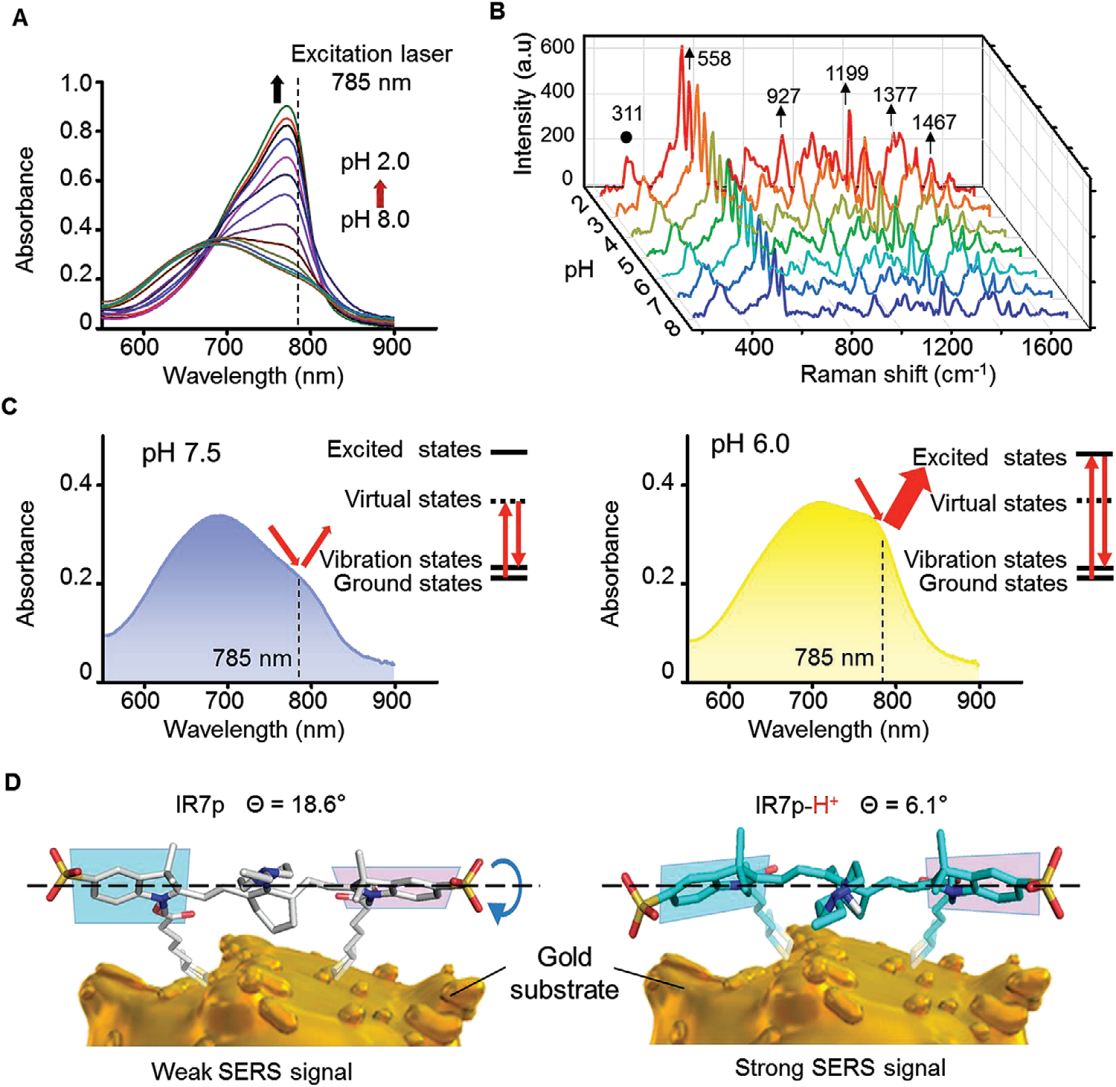
Proposed mechanisms of the pH responsiveness of the surface‐enhanced Raman scattering (SERS) chip. A) pH‐dependent absorption of IR7p (20 × 10^−6^ m). The maximum absorption wavelength shifted from 685 to 772 nm upon acidification. B) pH‐dependent SERS spectra of IR7p. While the peak at 311 cm^–1^ barely changed, other peaks increased with different degrees during acidification. C) The increased absorbance at 785 nm under acidic conditions triggered the resonance Raman scattering effect. D) The torsion angle between the two indolenium ring planes in IR7p decreased from 18.6° to 6.1° in the acidic environment.

The Raman reporter IR7p showed pH‐dependent absorption (**Figure** **3**A). With acidification from pH 8.0 to 2.0, the maximum absorbance of IR7p redshifted from 685 to 772 nm, and the maximal absorbance (772 nm) was 3.97 times higher than that (685 nm) measured at pH 8.0. Fingerprint‐like Raman spectra were collected by a handheld Raman scanner equipped with a 785 nm laser after dropping phosphate‐buffered saline solutions on the SERS chip (Figure 3B). The SERS chips showed homogeneity for pH measurement (Figure S4, Supporting Information). The peak at 311 cm^–1^ was attributed to the out‐of‐plane bending vibration of alkyl C−C bonds in the cyclohexene ring. Other major Raman peaks were assigned to chemical bonds in the *π*‐conjugated system (Figure S5, Supporting Information). The strong twin peaks at 527 and 558 cm^–1^ were attributed to the C−C out‐of‐plane bending of indolenium rings. The peaks at 927 and 1377 cm^–1^ were assigned to the C−H out‐of‐plane bending and C−H in‐plane bending of the olefin chain. A sharp peak at 1199 cm^–1^ was attributed to the C−C stretching of the olefin chain. The peak at 1467 cm^–1^ represented C−H bending of indole rings.^[^ ^25^ ^]^ Interestingly, while the peak at 311 cm^–1^ remained almost unchanged, other major peaks increased 2.6–3.4 times with acidification. The acidity‐responsive Raman signal enhancements could be explained by two mechanisms. First, the approach of the IR7p absorption maximum to the excitation wavelength (785 nm) of laser light triggers the resonance Raman scattering (RRS) effect, which increases the Raman intensity because the energy required for electron transition is close to that of the excited photon (Figure 3C). In the case of RRS effect, electrons transition from the ground states to the excited states and then return to the vibrational states. The intensity of Raman scattering light produced by this way is much higher than that of spontaneous Raman pathway, that is, electrons transition from the ground states to the virtual states and then return to the vibrational states. In contrast, the RRS effect did not affect the peak at 311 cm^–1^ because the energy needed for the *σ* to σ* transition was much higher than that of the excited photon (785 nm).^[^ ^26^ ^]^ Second, according to calculation of the lowest‐energy conformations (Figure 3D), the coaxial twist angle between the two indole planes decreased from 18.6° in IR7p to 6.1° in protonated IR7p (IR7p‐H^+^). The protonation‐triggered intramolecular indolenium ring rotation increased the coplanarity of the *π*‐conjugation system and led to Raman signal enhancements by increasing the Raman scattering cross‐section.^[^ ^27^ ^]^ Notably, the enhancement amplitudes of the Raman peaks were different. For example, the intensity of the peak at 1199 cm^–1^ increased by a factor of 3.4 when the pH decreased from 8.0 to 2.0, while only a 2.6‐fold enhancement was observed for the peak at 558 cm^–1^. The above phenomenon could be explained by the exclusive electron transition energy and oscillator strength of each Raman peak in IR7p. The stretching vibrations of the C−C bonds (1199 cm^–1^) in the olefin chain best matched the energy of the incident laser (785 nm) and therefore demonstrated the greatest resonance intensity enhancement.

### 2.3 Deep Learning Model Determines Sample's pH

**Figure 4:**
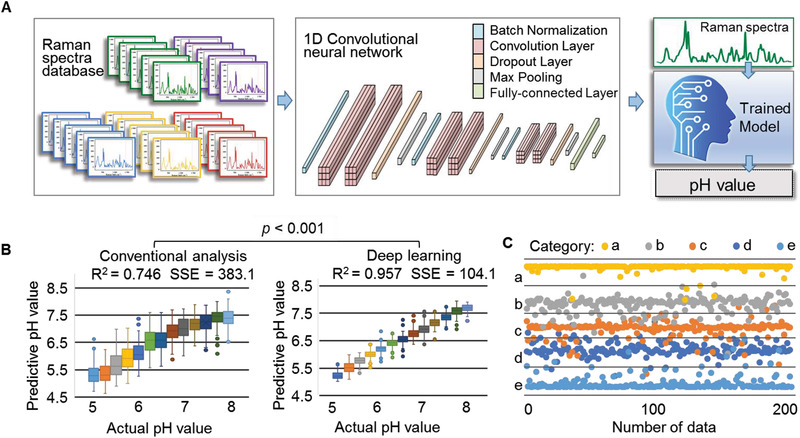
Deep learning model determines sample pH. A) Schematic diagram of the deep learning model. A total of 10361 Raman spectra from five groups of surface‐enhanced Raman scattering (SERS) chips were used to train the deep learning analysis system. B) Deep learning model significantly improved pH determination results compared to the traditional nonlinear regression model. C) The deep learning model automatically distinguished the data generated from different groups of SERS chips (five colors representing five groups of the SERS chips), which indicated the good robustness of the deep learning model.

Standard solutions with pH values ranging from 5.0 to 8.0 were dropped onto the SERS chip, and their Raman spectra were recorded. In total, 40 SERS chips were used to build a database containing 10361 Raman spectra, as shown in **Figure** **4**A. Each Raman spectrum was preprocessed to generate a vector corresponding to a pH value tag and a chip class label. A lightweight one‐dimensional convolutional neural network (CNN)^[^ ^28^ ^]^ was used to learn the intrinsic relationship between Raman spectra and their corresponding pH and chip labels. The network consisted of six convolution layers, three pooling layers, three batch normalization layers, three dropout layers, and two fully connected layers. The convolution layers were used to extract features from Raman spectra. The batch normalization layer was added before each convolution to normalize the data to accelerate the convergence speed of the model.^[^ ^29^ ^]^

The Raman spectrum database was randomly divided into a training set and testing set, with 80% as the training set containing 8289 spectra and 20% as the testing set containing 2072 spectra. The predicted results of the testing set are shown in a box plot (Figure 4B), where the abscissa represents the pH values of the samples measured by a pH meter and the ordinate represents the pH values predicted by the deep learning model. The deep learning model can determine the pH value with an accuracy of ±0.14 unit in the pH range of 6.0–7.5. Traditional nonlinear regression was used as the control method for pH prediction. **Table** **1** summarizes the pH prediction results between the deep learning model and the nonlinear regression model. Four numerical indexes, including the mean absolute error (MAE), standard deviation of MAE (SD), coefficient of determination (R^2^), and sum of the squared errors (SSE), were used to compare the differences between these two models. Compared with the classical regression method, the deep learning model made average improvements in MAE, SD, R^2^, and SSE of 45.9%, 53.6%, 28.3%, and 72.8%, respectively. Under the assumption that the two models are consistent, the model comparison passed the *t*‐test with *p* < 0.001, indicating that the deep learning model produced statistically better pH prediction than the traditional model. To verify the adaptability and robustness of our model to different chips, all chips were classified into five categories (Figure 4C). Points of five different colors represent data from the five categories of chips. The samples were automatically divided into intervals corresponding to the category, which means that the deep learning model can adapt to spectral data from different categories of chips, identify its category attributes, and eliminate the interference caused by the differences between chips. Therefore, using a deep learning model can obtain accurate and robust pH value estimation without the need to distinguish the differences among SERS chips.

**Table 1:** Comparison of the pH prediction results between the deep learning model and traditional linear regression

pH	MAE		SD		SSE	
	Deep learning	Regression	Deep learning	Regression	Deep learning	Regression
5.00	0.26	0.37	0.15	0.33	11.94	32.74
5.25	0.27	0.28	0.16	0.23	13.18	17.86
5.50	0.27	0.38	0.14	0.30	12.70	31.52
5.75	0.23	0.40	0.13	0.30	9.42	33.63
6.00	0.19	0.33	0.12	0.30	6.86	26.95
6.25	0.16	0.45	0.10	0.31	4.43	39.40
6.50	0.11	0.29	0.10	0.24	2.85	18.91
6.75	0.12	0.31	0.09	0.21	2.98	18.31
7.00	0.15	0.32	0.12	0.22	4.74	20.05
7.25	0.17	0.25	0.12	0.22	5.59	14.87
7.50	0.18	0.34	0.12	0.27	5.99	25.36
7.75	0.18	0.40	0.15	0.36	7.30	38.21
8.00	0.32	0.63	0.15	0.31	16.11	65.30
Average	0.20	0.37	0.13	0.28	8.01	29.47

MAE: mean absolute error, SD: standard deviation, SSE: sum of the squared errors.

### 2.4 SERS Navigation System Determines the pH of Tissue Mimics

**Figure 5:**
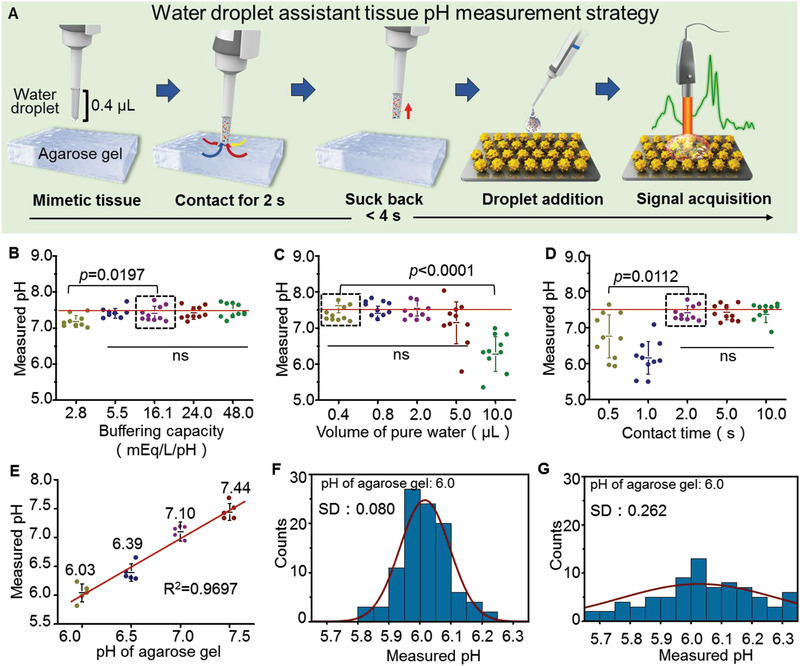
The surface‐enhanced Raman scattering (SERS) system determines pH on the mimetic tissue. A) Schematic diagram of measuring pH on an agarose gel that mimics brain tissue. The gels were developed with buffer solutions (pH 7.4) with similar compositions to intercellular fluid. When a pipette with a certain volume of ultrapure water was in contact with the surface of the mimetic tissue for a few seconds, the solute on the mimetic tissue dissolved into the ultrapure water. The water droplet was transferred to the SERS chip to acquire the Raman spectra. The functions of buffering capacity (sampling volume: 0.4 µL, tissue contact time: 2 s) (B), sampling water volume (tissue contact time: 2 s) (C), and tissue contact time (sampling volume: 0.4 µL) (D) on the pH measurement accuracy. E) The accuracy of the SERS system in determining physiological pH values (contact time: 2 s, sampling volume: 0.4 µL). The data were analyzed by unpaired *t* test and one‐way analysis of variance (ANOVA). *F* tests to compare variances were performed before statistical analysis. The uniformity of pH determined by single (F) and multiple SERS chips (G). ns: no significant difference.

The feasibility of this SERS system was first tested in agarose gels mimicking brain tissues (**Figure** **5**A). A new water droplet‐assisted tissue pH (WDA‐pH) measurement strategy was developed to determine pH values on tissue surfaces that are difficult to measure by conventional pH meters. In this strategy, an ultrapure water droplet contacted the agarose gel surface for 2 s and was then sucked back and dropped on the SERS chip. The SERS spectra of the aqueous samples were collected immediately and then processed by the homemade deep learning model. As shown in Figure 5B, the media in agarose gels with a buffering capacity above 2.8 mEq L^−1^ pH^−1^ showed satisfactory pH measurement accuracy. The effects of water droplet volume, contact area (Figure S6, Supporting Information), and contact time between the water droplet and agarose gel on the pH measurement accuracy were further investigated. Water droplet volumes ranging from 0.4 to 5.0 µL had no significant influence on the pH accuracy (Figure 5C). Notably, accurate pH determination was achieved when the contact time between the agarose gel and water droplet was longer than 2 s (Figure 5D). According to the above experimental results, the measurement parameters for in vivo studies were optimized to a sampling water volume of 0.4 µL and a tissue‐water droplet contact time of 2 s to achieve pH determination with high accuracy and speed. As shown in Figure 5E, the pH measurement discrepancies between the SERS system and pH meter were within 0.22 pH units. Furthermore, the reliability of determining the pH of samples on different locations of the SERS chip was evaluated by dropping the aqueous samples at different positions on a SERS chip. For 100 tests, the pH values of agarose gel (pH 6.0) were determined in a range of 5.95–6.05, and the standard deviation (SD) was 0.080 (Figure 5F). Moreover, the discrepancies among the SERS chips were further assessed for 10 randomly selected chips, and 10 pH measurements were performed on each chip. The narrow distribution pattern (SD: 0.262) of the determined pH values indicated small discrepancies among the SERS chips (Figure 5G).

### 2.5 Glioma Surgery Guided by Intraoperatively Locating Metabolic Acidity

**Figure 6:**
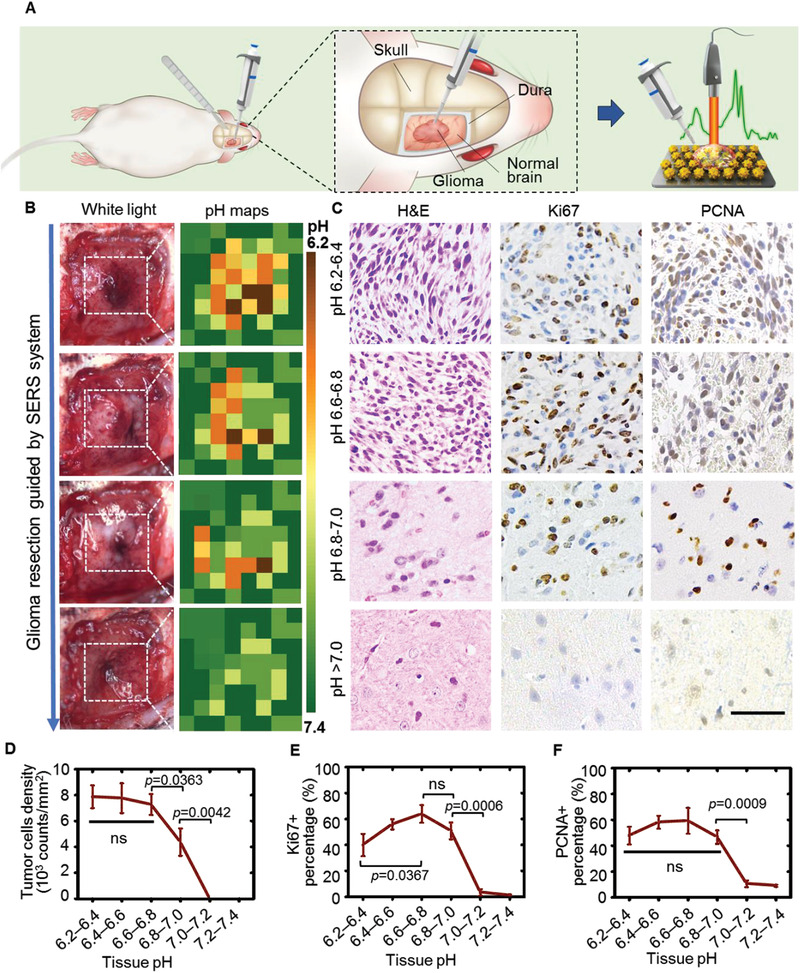
Acidic margin‐guided glioma surgery in animal models. A) Illustration of brain tumor surgery guided by the surface‐enhanced Raman scattering (SERS) system in model rats bearing orthotopic C6 glioma allografts. A pipette with an ultrapure water droplet (0.4 µL) on its tip was placed in contact with the suspicious tissue for 2 s, and then the samples were dropped onto the SERS chip to analyze the pH. B) Glioma resection with the guidance of a pH map of tumor cutting edges. The pH maps (right panel) of the tumor cutting edges (indicated by dashed square in the white light photographs, left panel) were generated intraoperatively by the SERS system. The surgery did not stop until all the regions with pH values below 7.0 were excised. C) Representative H&E, Ki67, and PCNA staining images of the excised tissues with different measured pH values. Scale bar: 50 µm. Tumor cell density (D), Ki67+ rate (E), and PCNA+ rate (F) of the excised tissues as a function of pH (*n* = 3). Unpaired *t* test for comparison between two sets of data and one‐way analysis of variance for comparison among three or more sets of data. The homogeneity of variance was tested before statistical analysis. ns: no significant difference.

Rat models bearing orthotopic C6 glioblastoma allografts were randomly divided into three groups (*n* = 5). In Group 1, the tumors were excised with reference to intraoperatively generated pH maps. In Group 2, the tumors were resected according to the preoperative MR images. In Group 3, the tumor‐bearing animals did not undergo any interventions. The general surgical procedure is described in **Figure** **6**A. After the craniotomy, the water droplet in the pipette tip was placed in contact with the suspicious tissues for 2 s and then dropped on the SERS chip. The Raman spectra of the water droplet were acquired by a handheld Raman scanner followed by pH determination via the deep learning model. By continuously collecting samples on the tumor cutting edge with the concomitant pH reading, a pH map (8 × 8 pixel) of the inspected tissue (1.0 × 1.0 cm) was generated within approximately 6.0 min (Figure 6B). Significantly, the acidic regions were distributed heterogeneously and predominately located in tumor margins. With guidance of this pH map, the tissue with the highest acidity was first excised, and the resection did not stop until no foci with pH < 7.0 were detected. Histological H&E staining of the excised tissues showed that a high density of cancer cells was observed in the tissues with pH values in the range of 6.2–6.8. In contrast, almost no cancer cells were found in the tissues with pH values above 7.0 (Figure 6C). Significantly, the immunoactivities of Ki67 and proliferating cell nuclear antigen (PCNA), representing the degree of cell proliferation, were evident in the tissues with pH values of 6.2–6.8 but barely detected in tissues with pH values above 7.0. Notably, while 6000–9000 cancer cells per square millimeter were found in the tissues with pH values ranging from 6.2–6.8, cancer cell density decreased sharply to 3000–5000 cells per square millimeter at pH 6.8–7.0 (Figure 6D). Remarkably, cancer cells were hardly detected in tissues with pH values above 7.0. A similar pH‐dependent cell proliferation rate is illustrated in Figure 6E,F. While the percentages of Ki67+ and PCNA+ cells remained above 40% in a pH range of 6.2–7.0 with a maximum (≈60%) at pH 6.6–6.8, the values decreased rapidly to less than 5% in tissue with pH above 7.0.

### 2.6 Acidic Margin‐Guided Strategy Improves Surgical Prognosis

**Figure 7:**
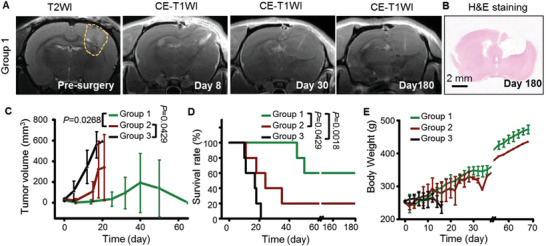
Acidic margin‐guided strategy improves surgical prognosis in animal models. A) Representative T2W‐ and contrast‐enhanced (CE) T1W‐MR images of model rats bearing C6 glioma allografts before and at selected days post acidic margin guided surgery. The tumor is indicated by yellow dash line. The mass at tumor bed after surgery was verified as hydrocephalus instead of the relapsed tumor tissue. B) Representative H&E staining images of rat brain sections at the end of the experiment. Scale bar: 2.0 mm. Tumor volume (C), survival rate (D), and body weight (E) of rat models after different treatments (*n* = 5 rats/group). An unpaired *t* test was used to compare the difference in tumor volume in each group, and the Gehan–Breslow–Wilcoxon test was used to compare the survival rate difference. The time points of statistical comparison in Figure 7C are day 25 in Group 1 versus day 21 in Group 2 and day 15 in Group 2 versus day 12 in Group 3. Group 1: Surface‐enhanced Raman scattering (SERS) system guided surgery, Group2: Gd‐DTPA guided surgery, and Group 3: no surgery.

The therapeutic efficacies were evaluated by contrast‐enhanced T1W MRI (**Figure** **7**A). Markedly improved prognosis was demonstrated in Group 1. No tumor recurrence was observed in more than 50% of animal models at 180 days post the surgery. H&E staining of rat brain sections at the end of the experiment verified the MRI results (Figure 7B, S7, Supporting Information). Figure 7C shows the tumor volume change as a function of time. For Group 3, tumor volume increased exponentially from 27.7 mm^3^ on day 0 to 534.6 mm^3^ on day 17. Even though the growth rate was flatter on days 17–20, the animal models demonstrated abnormal behaviors, including insensitivity to external stimuli, inability to maintain balance, and severe body weight loss. These models had to be sacrificed on days 16 to 21 according to animal ethics regulations. In Group 2, tumor relapse with rapid volume enhancement was found in 80% of animals, and these animals had to be sacrificed on days 18–24. In Group 1, tumor recurrence was not detected until 1 month post the surgery. Figure 7D shows the time‐dependent survival of the three groups. The first animal death in Group 3 was observed on day 10, and all the animals died in 21 days. A much flatter survival rate decrease was observed in Group 2, and 20% of animals survived for more than 40 days. Remarkably, the first animal death was not observed until 40 days in Group 1, and 60% of animals remained free of tumor relapse, with the survival rate remaining above 60% for more than 180 days. In addition, acidity‐guided surgery did not lead to obvious body weight loss after surgery (Figure 7E). In contrast, severe weight loss was observed in the late stages of tumor progression in Group 2 and Group 3.

### 2.7 Acidity Correlated Tissue Malignancy in Glioma Patients

**Figure 8:**
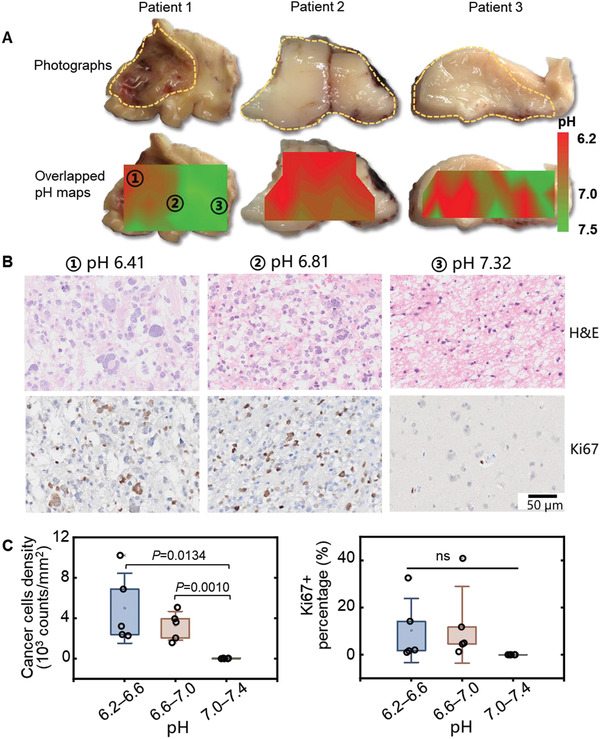
Acidity correlated malignancy found in excised tissues from glioma patients. A) White light photographic images and pH maps of intraoperatively excised tumor slides from three glioma patients. Tumor margins were marked by yellow dotted lines. B) H&E and Ki67 staining images of the regions with different measured pH values in the excised brain tissues. C) Density (H&E) and proliferation rate (Ki67+) of the cancer cells as a function of pH value determined by surface‐enhanced Raman scattering (SERS) system (*n* = 5 patients).

We next tested the feasibility of this SERS navigation system to define acidic margins in the excised tissues from glioma patients. **Figure** **8**A demonstrated the photographic images and pH map of the intraoperatively excised tissues from the glioma patients. Notably, acidification was demonstrated in tumor tissue but not in the neighboring non‐malignant tissues. The pH map generated by the SERS navigation system clearly demarcated tumor infiltrated regions that were verified by histological H&E staining (Figure S8, Supporting Information). The pH of the glioma tissues from the patients were measured between 6.2 and 6.8 in contrast to the nonmalignant brain tissues with the values from 7.0 to 7.5. Significantly, nuclear atypia, pleomorphism, dense cellularity, and microvascular thrombi were observed in H&E images of acidic tissues (Figure 8B). In contrast, above pathological characteristics were not observed in the tissue with pH above 7.0. Ki67 is a nuclear protein that is widely used as a marker of proliferating cells. The percentage of Ki67 positive cells in the acidic tissues was remarkably higher than that in tissues with neutral pH. Notably, tissue acidity correlated cancer cell density and proliferation level were demonstrated by analyzing the excised tissues from five glioma patients (Figure 8C). The cancer cell densities were determined as 5000, 3255 and 19 mm^−2^, respectively, in tissues with pH 6.2‒6.6, pH 6.6‒7.0, and pH 7.0‒7.4. Meanwhile, the Ki67+ cell percentages were measured as 10.3%, 12.7%, and 0%, respectively, for the tissues within above pH ranges.

## 3 Discussion

Metabolic reprogramming is one of the most striking features of tumor by which metabolic pathways are altered to meet the growth demand of cancer cells. The Warburg effect, a preference for glycolysis with metabolic products including lactate and H^+^, is a well‐known example of metabolic reprogramming in many types of tumors. Extracellular acidification shows multiple advantages as a biomarker in defining tumor‐infiltrated margins. First, extracellular acidification is a hallmark of solid tumors regardless of their phenotypes and genotypes. Second, extracellular acidity can be assessed noninvasively without tedious sample preparation. Third, due to the positive correlation between acidity and tissue malignancy, pH determination helps surgeons balance benefit and risk by evaluating malignancy degree of the suspicious tissues. Therefore, visualization of metabolic acidity provides a new opportunity to identify malignant tissues with high accuracy and velocity.

Recently, van Dam et al. reported a nanoprobe that successfully delineated the positive margins of different tumors in patients.^[^ ^30^ ^]^ This pH‐responsive nanoprobe intraoperatively visualized occult cancer and satellite lesions that could not be detected by visual inspection and palpation. Although this study verified extracellular acidity as a biomarker for guiding tumor surgery in patients, the nanoprobe might not be approved quickly by the drug administration authority due to the prerequisites to obtain quality assessment, safety, and efficacy profiles.^[^ ^31^ ^]^ Therefore, acidity‐guided strategies without the necessity to inject exogenous probes are desired. As a label‐free strategy, MS technologies, including desorption electrospray ionization (DESI),^[^ ^11^ ^]^ intelligent knife,^[^ ^32^ ^]^ and MasSpec Pen,^[^ ^33^ ^]^ have attracted attention due to their abilities to delineate positive margins by analyzing tumor‐associated lipid compounds. In comparison to MS technique, the SERS diagnostic system shows the advantage to determine extracellular acidity, the most abundant tumor associated metabolites. Additionally, the instrumental and operating cost of the SERS system is expected to be more affordable, which will accelerate its application in community hospitals.

The pH responsiveness of the SERS chip stems from the Raman reporter molecule IR7p, which shows multiple advantages. First, the absorbance maximum of IR7p perfectly overlaps with the wavelength of incident laser light, which triggers the SERRS effect. The sensitivity of the SERRS signal can be increased by 1–2 orders of magnitude in comparison to the SERS signal.^[^ ^19^ ^]^ Second, the *π*‐conjugated system in IR7p is much more complex than conventional Raman reporter molecules such as 4‐mercaptobenzoic acid (4‐MBA),^[^ ^34^ ^]^ which not only offers the SERS chip with characteristic fingerprint‐like Raman spectra but also provides an unprecedented opportunity for pH determination via a deep learning model. Nanostars were chosen as the metallic substrate of the SERS chip for the following reasons. (1) The multiple gaps between the nanostar branches generate numerous “hotspots,” which significantly increase the sensitivity (Figures S9 and S10, Supporting Information). (2) The star‐shaped nanoparticle morphology redshifts the localized surface plasmon resonance (LSPR) to the NIR wavelength range, which triggers the SERRS effect^[^ ^20^ ^]^ (Figure S3, Supporting Information). Traditional machine learning algorithms, such as regression analysis, usually require predefined input variables to establish a model between the input variables and pH values from the current measurements of SERS chips. However, regression analysis does not adaptively adjust the input variables and their combinations when deviations occur between SERS chips. In contrast, the deep learning method can automatically filter useful variables from the spectrum, and the method can be used to predict any pH value in any chip as long as the corresponding data are available in the training set.

Rapidly and accurately delineating the pH values of tumor cutting edges is a prerequisite for acidic margin‐guided surgery. To achieve the above goal, we proposed the WDA‐pH strategy, by which the acidity of the tissue surface could be measured by using a pure water droplet as a transfer medium. The advantages of the WDA‐pH strategy are: (1) measurement of pH on the exposed tissue surface, which cannot be accessed by electrode‐based pH meters; (2) minimized damage to suspicious tissues via nondestructive sampling method; (3) flexibility in choosing the measurement location, which avoids regions polluted by leaked blood, cerebrospinal fluid, or other contaminants. The sampling procedure was optimized to increase pH measurement speed. For example, the contact time between the water droplet and tissue surface was optimized to 2 s. Notably, considering the time for Raman signal acquisition and data processing, the pH value of a sample could be determined in as short as 4 s. By this way, a pH map of a tissue area of 2 cm^2^ could be generated in 4 min (spatial resolution: 2.0 mm). Compared to the conventional pH meter, the SERS navigation system shows its advantages to determine the pH values of the suspicious tissues for below reasons. First, for tissue pH measurement, the electrode has to insert into the brain tissue. The tissue injury is neurosurgeons trying to avoid. Second, microcapillary pH meter cannot measure the samples with volume less than 10 µL, which is far beyond the sample volume (0.2–0.4 µL) collected by the SERS system. Third, the SERS system can continually read pH values of multiple samples with an interval of 4 s, which is much faster than a conventional pH meter by considering the time cost for electrode washing and recalibration.

Accurately determining the acidity of the suspicious tissue is prerequisite for the successful application of the WDA‐pH strategy. The buffering capacity of tumor intercellular fluid was determined in a range of 5–60 mEq L^−1^ pH^−1^,^[^ ^35^, ^36^ ^]^ which is much higher than the threshold of 2.8 mEq L^−1^ pH^−1^ for WDA‐pH strategy. Under the condition of room temperature and normal atmosphere, the concentration of CO_2_ in exposed water is about 0.012 × 10^−3^ m.^[^ ^37^ ^]^ According to the dissociation constant of carbonic acid (*K*a_1_: 4.45 × 10^–7^, *K*a_2_: 4.69 × 10^–11^), its buffer capacity is calculated about 0.0059 mEq L^−1^ pH^−1^. Therefore, potential interference induced by the dissolved CO_2_ in the water droplet can be ignored. The blood leakage indeed disrupts the pH measurement due to the high buffering capability of plasma (16.1 mEq L^−1^).^[^ ^38^ ^]^ However, our experiment showed that the interference can be minimized by timely wipe the blood contamination with absorbent gauze (Figure S11, Supporting Information).

## 4 Conclusion

In this work, we develop a SERS navigation system intraoperatively delineating the acidic regions of glioma. This SERS system helps to accelerate the clinical translation of acidic margin‐guided surgery by avoiding the administration of exogenous imaging probes. Considering that intercellular acidification is a hallmark of solid tumors, this SERS system holds the promise for the treatment of solid tumors with an infiltrative nature. Due to the correlation between tissue acidity and malignancy, this SERS system enables the surgeon to quantitatively evaluate tissue malignancy, which will be helpful to balance benefit and risk in excising the tissues around eloquent regions.

## 5 Experimental Section

### Chemicals and Materials

HAuCl_4_·4H_2_O, Sodium citrate·2H_2_O, aminopropyl triethoxy silane (APTES), NH_3_·H_2_O, 4‐(2‐hydroxyerhyl) piperazine‐1‐erhanesulfonic acid (HEPES), polyoxymethylene, chloral hydrate, and glass cover slips were purchased from General Reagent, Shanghai. The 4‐inch diameter silicon wafers were purchased from Shanghai Zhiyan Electronic Technology Co., Ltd. H_2_O_2_ (30%), Na_2_HPO_4_·12H_2_O (99.0%), citric acid·H_2_O (99.0%), and HCl (37.5%) were purchased from Sinopharm chemical reagent Co., Ltd. Ultrapure (Up) water was produced by a MT system (18.2 MΩ cm, Shanghai Leading Water Treatment Equipment Co., Ltd., China). All solvents were obtained from commercial sources and used without further purification.

### Fabrication of SERS Chips

The SERS chips were synthesized as the following. Silicon wafer (24 × 50 mm) was cleaned in the solution of H_2_O:NH_3_·H_2_O:H_2_O_2_ (5:1:1) at 80 °C for 30 min in order to expose hydroxyl on the surface. After washed by ethanol for three times, the wafer was functionalized with 2% 3‐aminopropyl triethoxy silane (APTES) in ethanol at room temperature for 12 h and then heated at 100 °C for 2 h. The gold nanospheres were synthesized by Frens’ method with a little modification.^[^ ^39^ ^]^ In brief, 1.0 mL of 1% trisodium citrate dihydrate and 1.0 mL of 24 × 10^−3^ m HAuCl_4_ aqueous solution were added into the 98 mL boiling ultrapure water. After 3 min, the same amount of HAuCl_4_ and trisodium citrate dihydrate were added into the reaction system at an interval of 40 s to synthesize the 45 nm gold nanospheres. The amino‐functionalized silicon wafer was immersed in the gold gel to adsorb the gold nanospheres for 48 h and then immersed in the 30 mL of 70 × 10^−3^ m HEPES and 0.5 × 10^−3^ m HAuCl_4_ aqueous solution for 90 min at 10 °C to grow up short branches. The SERS substrate prepared as above was immersed in the 25 × 10^−6^ m IR7p dissolved in methanol for 6 h and washed with methanol three times.

### Absorption Studies

The pH‐dependent absorption spectra of IR7p were acquired by mixing 100 µL of 200 × 10^−6^ m methanol solution and 900 µL of buffer solution containing disodium Na_2_HPO_4_ and citric acid. The buffer saline solution containing the same proportion of methanol (10%) was used as control.

### Raman Spectroscopy Studies

To obtain the SERS spectra under pH 5.0–8.0, 2 µL buffer solutions of different pH from 5.0 to 8.0 were dropped onto the dry SERS chip. The Raman spectra of water drop position were collected by using an Ocean Optics QE65 Pro handheld Raman scanner with a 785 nm excitation laser (350 mW), grating of 600 gr mm^−1^, and acquisition time of 500 ms. The studies of the effects of contact time, ultrapure water volume, and buffer capacity were performed with the same Raman spectra acquisition parameter above. The sample extracting process is also the same as described in experiment part of “Acidic margin‐guided intraoperative glioma resection.”

### Development of SERS Spectra Database

The Raman spectral database contains 10361 spectra from 40 chips. The 40 chips were prepared under the same preparation conditions such as growth time, temperature, and concentration. SERS spectra of standard buffer solution of different pH from 5.00–8.00 with an interval of 0.25 were collected on each chip, and 20 spectra were collected for each pH value, that is, about 260 spectra per chip. For a same chip, the *I*_527_/*I*_331_ of the Raman spectroscopy showed an obvious linear correlation with the pH value. Standard solution with a pH of 6.50 was used to test 40 chips. According to the difference (err = calculated pH–real pH) between the pH value calculated by the Raman spectra of the corresponding chip and the real value 6.50, the 40 chips were divided into five groups: a. err > 0.5, b. 0.1 < err < 0.5, c. −0.1 < err < 0.1, d. −0.2 < err ← 0.1, e. err ← 0.2. The purpose of grouping was to verify the robustness of traditional linear regression and deep learning algorithms in pH prediction.

### Establishment of Raman Deep Learning Model

For each Raman spectrum data preparing for deep learning model training, the area of peak 1 at 331 cm^–1^ was calculated. In detail, The Raman shift of the right trough of Peak 1 is in the range of (360, 390), Then the program looked for the lowest points x_2_ in these two ranges, respectively. After that, the program made a vertical line to the *Y* axis through the point (x_2_, y_2_) and found the point intersecting with the image as the left endpoint of peak 1. The area of peak 1 is integrated from x_1_ to x_2_. Then the peak 1 would be removed from the original spectrum after the area was obtained. Raman shift of more than 1700 cm^–1^ in the spectrum was also removed since no available information was contained. Therefore, we only kept the 80th to 335th points of the original Raman spectra, and finally get the new data for training the deep learning model. Each data also had a corresponding label, including pH and category labels. We built the model based on one‐dimensional convolution neural network. The network consists of six convolution operations, which were used to extract features from Raman spectra. The Batch‐Normalization layer was added before each convolution to normalize the data to accelerate the convergence speed of the model. After every two convolutions, the dimension of features was reduced by a pooling layer. Redundant information was removed and the amount of computation was reduced. In addition, dropout layer randomly discarded the parameters to prevent over‐fitting and increase the generalization ability of the model. Finally, the data were fitted and regressed through the fully‐connected layers. After several iterations of training, the model automatically adjusted the parameters of each layer to make the output as consistent as possible with the label value corresponding to the input training data. The program of peak area ratio automatic calculating was realized by MATLAB 2019b. The program scanned the specified folder every 100 ms, and selected the latest spectrum file loading program.

### Reporter Molecule Conformation Prediction

The energy minimization was performed using full minimization module in Discovery Studio 2019. MMFF force field was applied. 1000 steps of Steepest Descent with a RMS gradient tolerance of 3 was performed, followed by Conjugate Gradient minimization. Other parameters were set as default.

### Establishment of Rat Models

Animal experiments were conducted according to the guidelines approved by the Ethics Committee of Fudan University School of Pharmacy (2019‐03‐FY‐LC‐01). Male Sprague Dawley rats (200–220 g, 5–7 weeks, Shanghai Sippr‐BK laboratory animal Co. Ltd., China) were used to establish the orthotopic Glioblastoma Xenograft models. The rat glioma cell line C6 was purchased from the American Type Culture Collection (Manassas, VA) and subcultured every other day at 37 °C with 5% CO_2_ in Dulbecco's modified Eagle's medium supplemented with 10% FBS penicillin (100 U mL^−1^). In brief, the rats were anesthetized by 10% chloral hydrate and fixed on the stereo positioning instrument firstly. C6 glioblastoma cells (5 × 10^5^ cells in 6.0 µL PBS) were injected into the striatum at 4.0 mm to the right side of the bregma and 4.7 mm deep into the brain surfaces. The surgeries were performed 8–10 days after the glioma models establishment.

### Preparation of Agarose Gel‐Based Tissue Memetics

The buffer solution of about 48.0 mEq L^−1^ pH^−1^ buffer capacity was prepared by mixing the solution of disodium hydrogen phosphate (0.2 m) and citric acid (0.1 m). The other buffer solutions (24.0, 16.1, 5.5, 2.8 mEq L^−1^ pH^−1^) were obtained by diluting 2, 3, 10, 20 times with ultrapure water, respectively. An amount of 100 mg of agarose powder was added into 10 mL of buffer solution. The mixture changed to gel after boiling for 10 s and cooling to room temperature.

### Acidic Margin Guided Glioma Resection

Rat models bearing orthotopic C6 glioblastoma xenografts were randomly divided into three groups including SERS chip‐guided surgery group, white light‐guided surgery group, and sham‐operation group. Each rat was anesthetized with 1 mL of 10% chloral hydrate by intraperitoneal injection. Then, the procedures of craniotomy including skin incision, muscle dissection, and skull drilling were performed sequentially. A skull window of about 1.5 × 1.0 cm was made finally. The brain was visible after dura mater, arachnoid mater and pia mater were cut off carefully. An amount of 0.4 µL of ultrapure water that had been newly boiled and cooled to 37 °C was contacted with tissue surface with a pipette for 2 s. The spatial profile of pH was obtained by the pipette fixing on the stereotaxic apparatus. Each pixel in the pH maps represented the rectangle areas of about 1.2  × 1.2 mm. The pipette was moved step by step to each position precisely by adjusting the *X*–*Y*–*Z* axis of stereotaxic apparatus. The ultrapure water with dissolved tumor metabolites was dropped onto the SERS chip and the Raman spectra of this place were collected at once. The Raman scanner was connected to a computer installed with OceanView software and the intelligent automatic processing program. The other acquisition parameter and machine were the same as that in SERS chip characterization study. The pH values of the samples were showed on the screen after the spectra collection and the calculation of Intelligent automatic processing program. The tissues with pH value lower than 7.0 were all removed.

### MRI Method

The prognosis evaluation of rat models was performed on an 11.7 T horizontal Bruker scanner (Bruker BioSpin, Billerica, MA). The rats were anaesthetized with 5% isoflurane (Pharmachem, Eagle Farm, Australia) before imaging and 2% isoflurane in 50% oxygen during imaging. Body temperature and breathing rate were monitored in real time by an animal life monitoring system (SAInstruments, Inc., Stony Brook, NY, USA). T2W‐MRI parameters areas are followings: repetition time (TR)/echo time (TE) = 4000/30 ms; acquisition matrix = 256 × 256, NEX = 2, field of view (FOV) = 32 × 32 mm; slice thickness = 0.5 mm; number of slices = 24, T1W‐MRI parameters are as followings: TR/TE = 1000/5.5 ms; acquisition matrix = 256 × 256; NEX = 2; FOV = 32 × 32 mm; slice thickness = 0.5 mm; number of slices = 24; T1value was measured using a rapid‐acquisition with relaxation enhancement sequence with 6 inversion time (5000, 2500, 1800,1200, 800, and 300 ms). A dose of 0.1 × 10^−3^ m Gd^3+^−diethylenetriaminepentaacetic acid (DTPA)/kg was administered via the tail vein 1 min before the acquisition of contrast‐enhanced T1W images.

### Pathological Section Studies

The whole brain or the tissues removed from the tumor were immersed in 4% paraformaldehyde (PFA) for 24 h and embedded with paraffin. The slices (thickness: 10 µm) were treated with dimethylbenzene, ethanol of different concentrations, and water in turn before hematoxylin and eosin (H&E) staining. For Ki67 and PCNA immunohistochemistry (IHC) staining, the antigen needed to be repaired with sodium citrate‐HCl solution. Then the anti‐ki67 and PCNA antibodies were dripped onto the slices and incubated in the second antibody, 3,3'‐diaminobenzidine (DAB) and hematoxylin successively. The tissue slides were observed and photographed under an optical microscope (Olympus BX51, Japan).

### Studies on Glioma Tissues from Patients

The experiment of glioma tissue samples from patients was carried out in accordance with the ethical regulations of Huashan Hospital Affiliated to Fudan University (No. 256, 2015). The informed written consent from all participants or next of kin was obtained prior to the study. The pH map of the excised tissue was delineated by the SERS navigation system in 10 min after tissue excision. The sampling and detection methods were as same as that used in the animal experiments. After collecting the pH map, the tissues were fixed with 4% paraformaldehyde and histologically studied by H&E and Ki67 staining. The demographic and clinical characteristic of the five Asian patients in this article were shown in Table S1 (Supporting Information).

### Statistical Analysis

Statistical analysis was performed using Origin 9.1 software (Microcal Software Inc., Northampton, USA), and GraphPad Prism 8.00. Data are presented as the mean ± S.D. for all results. Statistical differences between two groups were tested with a two‐tailed Student's *t* test. One‐way analysis of variance (ANOVA) was applied to the multiple comparisons. Survival curves were compared using Mantel‐Cos test. The body weight of rats was normalized to 250 g on day 0. Probability value (*p*‐value) less than 0.05 was considered significant.

## Conflict of Interest

The authors declare no conflict of interest.

## Supporting information

Supporting Information

## Data Availability

The data that support the findings of this study are available from the corresponding author upon reasonable request.
