# Supplementary material for: Intelligent SERS Navigation System Guiding Brain Tumor Surgery by Intraoperatively Delineating the Metabolic Acidosis
Source: Adv Sci (Weinh). 2022 Jan 12;9(7):2104935. doi: 10.1002/advs.202104935 (PMC8895125; doi:10.1002/advs.202104935)
Supplement: Supplementary file 1 — Supporting Information [file ADVS-9-2104935-s001.pdf]

ADVANCED SCIENCE
Open Access

# Supporting Information

for *Adv. Sci.,* DOI: 10.1002/advs.202104935

# Intelligent SERS Navigation System Guiding Brain Tumor Surgery by Intraoperatively Delineating the Metabolic Acidosis

*Ziyi Jin[+a], Qi Yue[+e], Wenjia Duan[+a], An Sui[+c], Botao Zhao[b], Yinhui Deng[c], Yuting Zhai[b], Yuwen Zhang[b], Tao Sun[a], Guang-Ping Zhang[d], Limei Han[a], Ying Mao*[e], Jinhua Yu*[c], Xiao-Yong Zhang*[b] and Cong Li*[a]*

# Supporting Information

## Intelligent SERS Navigation System Guiding Brain Tumor Surgery by Intraoperatively Delineating the Metabolic Acidosis

Ziyi Jin[+a], Qi Yue[+e], Wenjia Duan[+a], An Sui[+c], Botao Zhao[b], Yinhui Deng [c], Yuting Zhai[b], Yuwen Zhang[b], Tao Sun[a], Guang-Ping Zhang [d], Limei Han[a], Ying Mao*[e], Jinhua Yu*[c], Xiao-Yong Zhang*[b] and Cong Li*[a]

[a] Key Laboratory of Smart Drug Delivery, Ministry of Education, School of Pharmacy, Fudan University, Shanghai 201203, China. E-mail: congli@fudan.edu.cn

[b]Key Laboratory of Computational Neuroscience and Brain-Inspired Intelligence, Ministry of Education, Institute of Science and Technology for Brain-Inspired Intelligence, Fudan University, Shanghai 200433, China. E-mail: xiaoyong_zhang@fudan.edu.cn

[c]School of Information Science and Technology, Fudan University, Shanghai 200438, China. E-mail: jhyu@fudan.edu.cn

[d]School of Physics and Electronics, Shandong Normal University, Jinan, 250358, China.

[e]Department of neurosurgery, Huashan Hospital, Fudan University, Shanghai, 200040, China. E-mail: maoying@fudan.edu.cn

[+] These authors contributed equally to this work.

**Synthesis of Raman reporter molecules.** The detailed synthesis of IR7p can be found in our previous report[5]. In brief (**Fig S1**), compound a (100 mg, 0.58 mmol) and compound b (365 mg, 1.13 mmol) were dissolved in mixed solvent ($C_4H_{10}O$:$C_3H_6O$ = 7:3, V:V), and kept it refluxing with stirring under anaerobic condition overnight at 120 °C. The product was recrystallized in anhydrous ether. Bright green solid was further purified via silica gel chromatography ($CH_2Cl_2$:$CH_3OH$ =3:1, V:V) to give pure IR7. IR7 (200 mg, 0.256 mmol) was further dissolved in DMSO. To this solution, ethyl piperazine (300 μL, 2.33 mM) was added and followed by stirring for 12 h at 100 °C to afford compound c as a royal blue solution. The blue solution was further purified via silica gel chromatography ($CH_2Cl_2$:$CH_3OH$ = 2.5:1, V:V) to obtain pure c. Finally, compound c (45.0 mg, 0.0058 mmol) was mixed with DMAP (2.11 mg, 0.0172 mmol), lipoic acid (6.0 mg, 0.0288 mmol) and DCC (2.68 μL, 0.0172 mmol) in DMF. The reaction was kept in dark for 18 h. The solution was further purified via silica gel chromatography ($CH_2Cl_2$:$CH_3OH$= 5:1, V:V) to give pure IR7p as a dark blue solid.

**SEM studies.** SEM images were captured by ZEISS Gemini 500. The chips were pasted on the nail table by carbon conductive tapes. EHT=3.00 or 6.00 kV, WD=3.7 or 4.0 mm,

Mag=15.00/30.00/50.00/200.00 K X. Signal A= Inlens, Aperture Size=20.00 μm. The gold nanostars distributed on the silicon wafer uniformly. There were about $1.36\times10^{10}$ nanoparticles per square centimeter **(Figure S2).** The gold nanoparticles in the SERS chips growing for 20 min had some protuberances with height of 2–6 nm. Compared with that before growth, the diameter of nanoparticles also increased 10–15 nm.

**Extinction spectra studies.** The silicon wafers in SERS chips were replaced by the glass coverslips (24 mm×50 mm) in this study for the optical transparency of glass. The other fabrication parameters were the same. The extinction spectra were obtained by fixing the chips in the sample pool location in the spectrophotometer (UV2550, Shimadzu Co.). The clean coverslips were used as the control. The scanning wavelength range was 400–900 nm. The maximal extinction wavelength of the SERS chips of gold nanospheres was 529 nm, shorter than that of gold nanostars chips, whose maximal extinction wavelength was 608 nm. The increased of extinction in the range of 638 nm–900 nm and 475 nm–571 nm suggested that the molecule IR7p were successfully modified on the surface of gold nanostars **(Fig. S3)**.

**Raman spectroscopy studies**. No new peaks were observed by comparing the SERS spectra of IR7p to that of IR7p-pre. The main difference between the spectra of IR7p and IR7p-pre is that the SERS peak at 1097 $cm^{-1}$ and 1199 $cm^{-1}$ of IR7p is relatively stronger than that of IR7p-pre **(Fig S5).** Therefore, we attributed all the SERS peaks to the bond in the compound IR7p-pre.

The SERS substrate post different nanoparticle growth time were treated with the methanol solution of IR7p for 12 h. The SERS signal intensities of chips after IR7p modification were significantly stronger than that of chips without IR7p fabrication. The fitting curves of $I_{558}/I_{311}$ and pH value showed that the SERS chips with longer growth time have steeper trend, which is helpful to calculate pH more accurately **(Figure S9C and S9D)**.

The results of limit of detection (LOD) and peak assignment were obtained by using Ocean Optics QE65 Pro handheld Raman scanner. The power of 785 nm laser focused on samples are 350 mW and the acquisition time was 1 s. The LOD experiment was performed by dropping the methanol solution of IR7p onto the SERS chips (growth time: 90 min). The spectra were recorded by focusing the laser in the center of the droplet area. The signal to noise ratio (SNR) of LOD was measured as 3. The spectra of IR7p with the concentration higher than $10^{-5}$ M can be used to calculated pH accurately because the noise in this condition is weak enough to be ignored. We choose the IR7p concentration of $2\times10^{-5}$ M to carry out the follow-up pH measuring experiment **(Figure S10).**

The SERS signal homogeneity was investigated by dropping the buffer solution (pH 6.0,

2.0 μL) onto 10 spots across the SERS chip. Two similar experiments were performed by replacing the buffer solution with pH 6.5 and 7.0. Five spectra were acquired for each spot (excitation laser wavelength: 785 nm, excitation laser intensity: 350 mW, grating: 600 gr/mm, acquisition time: 500 ms). The experimental results were showed in **Figure S4**. Even though the Raman intensities collected at different locations of the SERS chip were diverse, the Raman intensity ratios between the Peak at 558 $cm^{-1}$ and Peak at 311 $cm^{-1}$ kept constant for all the spots. The standard deviations of the pH values were measured as 4.1%, 3.5% and 4.8% respectively for buffer solutions with pH 6.0, 6.5 and 7.0. Above experimental data indicated the satisfactory homogeneity of the SERS chip.

In order to study the effect of contact area on the pH measurement accuracy, four different pipette tips with diameters ranged from 469 μm to 956 μm were applied to extract samples from the mimetic tissue (agarose gel, pH 7.5). The mimetic tissue was fabricated as following. The buffer solution of about 48.0 mEq/L/pH buffer capacity was prepared by mixing the solution of disodium hydrogen phosphate (0.2 M) and citric acid (0.1 M). 100 mg of agarose powder was added into 10 mL of buffer solution. The mixture changed to gel after boiling for 10 seconds and cooling to room temperature. The diameter of tips was measured in microscope image (Olympus BX53, DP27) by the software Cellsens. The cross section of the tips is considered to be an ideal circle, and the contact area is calculated by the circle area formula. The tips containing 0.4 μL ultrapure water were contact with the agarose gel for 1 s or 2 s. Then the water was added to the SERS chip for Raman spectra acquisition. Sample extraction and pH detection procedure were carried out ten times for each kind of tips and 5 spectra were acquired for every Raman detection. When the contact time was 1.0 s, the average pH value measured by pipette tip with diameter of 469 μm was remarkably lower than the tip with contacting area diameter above 622 μm. Notably, tip area showed negligible effect to pH measurement if the contact time was longer than 2 s **(Figure S6).**

In order to study the effect of blood contamination on the pH measurement by the SERS system. Three pieces of mimetic tissue made of agarose gel (pH 6.0 and pH 6.5) were prepared **(Figure S11)**. The fresh blood from mice was dropped on the centre of agarose gel and cleaned with cotton swabs 5 s later. The pH was measured at the same location 20 s later (ultrapure water volume:0.4 μL, contact time: 2 s). The measured pH for agarose gel of pH 6.0 ,6.5 and 7.0 was 5.98 (SD: 0.146), 6.45 (SD: 0.150) and 7.13 (SD: 0.170) respectively. So we believe the blood contamination has little effect on the measured pH by SERS system.

**Finite difference time domain method**. The absorption spectra of nanostructures model were calculated using FDTD method by MIT Electromagnetic Equation Propagation (MEEP)

simulation software package[1-3] in 3D mode. The simulation region is inside a cuboid with dimension of 500nm ×500nm ×100nm, and a 50-nm-thick perfect matched layer is added at each face boundary. The resolution of the simulation in space region is 2Å and the corresponding Courant factor is 0.95. For the calculation electric field distribution and charge distribution, a monochromatic light source (781nm) was used. The primitivity of Au reported by Johnson and Christy in 1972[4] was used to describe optical properties of all the Au nano-structures. The FDTD results showed that the $|E/E_0|$ in the gap of two nanostars (maximum: 178.9) is much higher than that on the surface of nanospheres (maximum: 4.5) **(Figure S9A)** because of the "Hot Spots" effect.

**Pathological section studies.** The enlarged images of the pathological section on coronal plane of the rat brains were shown in the Supplementary Fig.4. The section images in Group 1 show that no recurrence was observed in this rat model bearing orthotopic glioblastoma allograft 180 days post the surgery guiding by intelligent SERS navigation system. The section images in Group 2 show the serious recurrence and infiltration after the surgery in Group2.

**Cell preparation**. The C6 cells for rat orthotopic glioblastoma allograft model establishment were purchased from American Type Culture Collection (Manassas, VA). Cells were maintained and subcultured every other day in Dulbecco's modified Eagle's medium supplemented with 10% FBS in Petri dish of 100 mm diameter (temperature: 37 °C; $CO_2$ concentration: 5%). Cell suspensions for the establishment of glioma models were prepared by the following methods. After the medium was removed and washed twice with PBS, 1 mL of 0.25% trypsin-EDTA was added to the sample for digesting the cells from the bottom of the dish. 2 mL DMEM culture medium was added to Inactivated trypsin-EDTA. The cell suspensions were then transferred to centrifuge tube and washed by centrifugation, supernatant removed, adding PBS and pipetting. The prepared cells were stored in an ice box for intracerebral injection.

Table 1. Demographic and clinical characteristic of five Asian patients.

| No. | Gender | Age | WHO grade | Pathological classification |
|---|---|---|---|---|
| 1 | Female | 28 | Ⅳ | Glioblastoma |
| 2 | Female | 41 | Ⅱ | Oligodendroglioma |
| 3 | Female | 57 | Ⅱ | Oligodendroglioma |
| 4 | Male | 53 | Ⅳ | Glioblastoma |
| 5 | Female | 28 | Ⅲ | Anaplastic Astrocytoma |

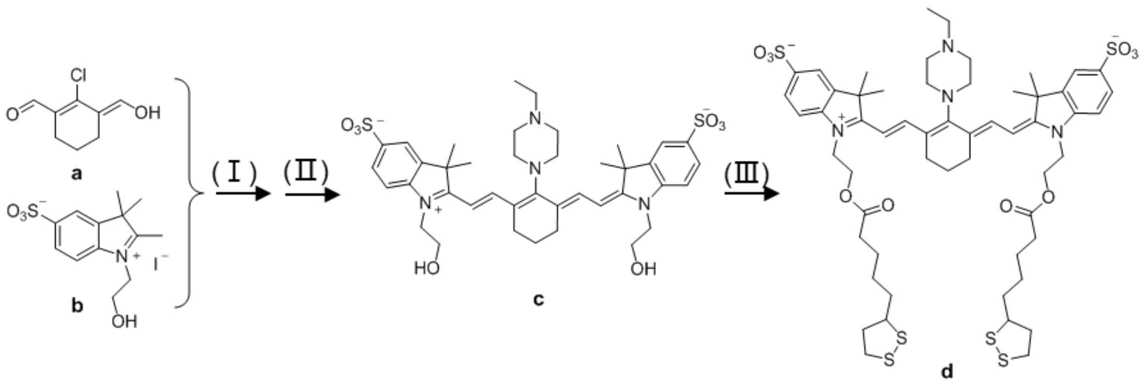

**Figure S1. The synthesis of IR7p.** (Ⅰ) n-BuOH/Toluene, reflux. (Ⅱ) ethyl piperazine, DMSO. (Ⅲ) Lipoic acid, DCC, TsOH, DCM.

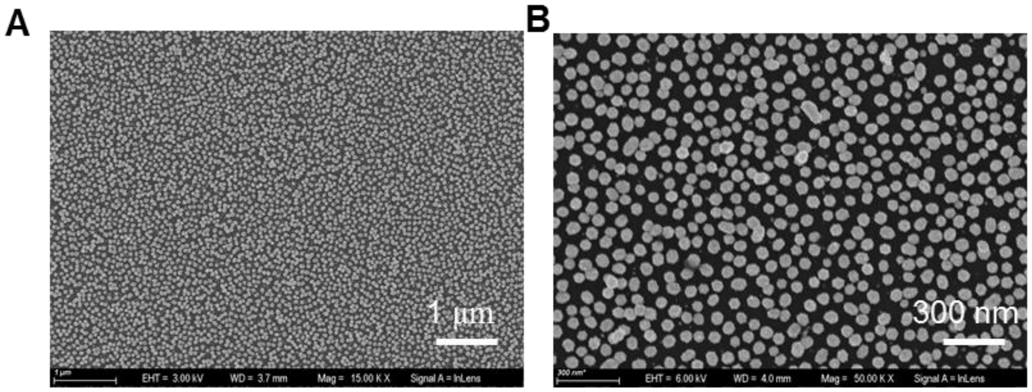


**Figure S2. SEM images of gold nanostars on the SERS chips.** (A) The SEM image with wide field of view of SERS chip (grown for 90 min), scale bar:1.0 µm. (B) The SEM image of SERS chip (grown for 20 min), scale bar: 300 nm.

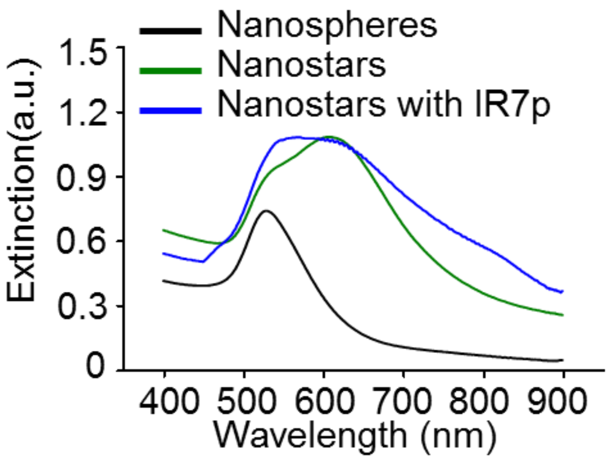


**Figure S3. The extinction spectra of SERS chip modified with nanospheres, nanostars and nanostars labeled with IR7p.**

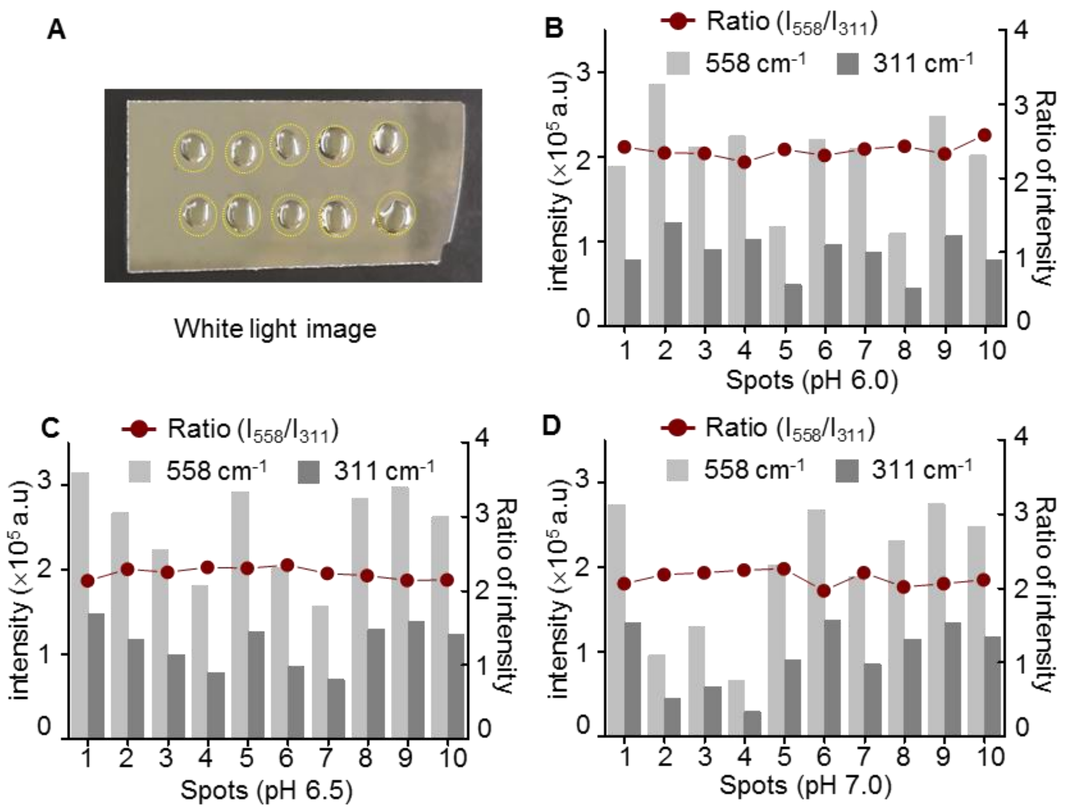


**Figure S4. Home-made SERS chips showed homogeneity for pH measurement.** (A) The white light image of ten buffer droplets placed at different locations on the SERS chip. (B, C, D) Measured intensities of the Raman peaks at 558 $cm^{-1}$ and 311 $cm^{-1}$ of the ten locations indicated in panel A (columns). The intensity ratio between peak 558 $cm^{-1}$ and 311 $cm^{-1}$ measured at ten spots on the SERS chip (curves).

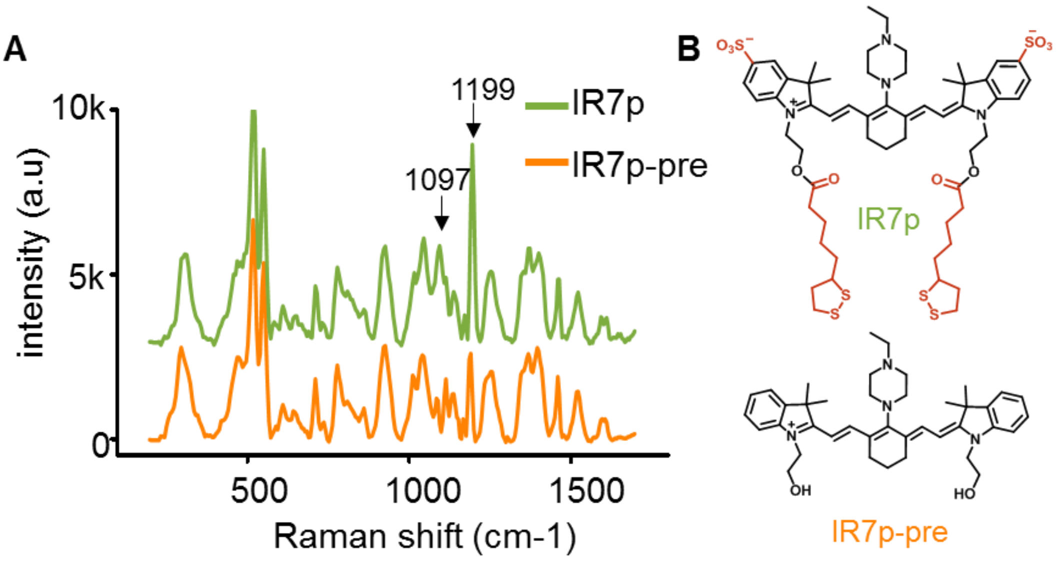


**Figure S5. SERS signals of lipoic acid and sulfonate groups are too weak to be detected.** (A) SERS spectra of IR7p and IR7p-pre on SERS chip. The peaks intensity at 1097 $cm^{-1}$ and

1199 cm$^{-1}$ were relatively stronger in IR7p spectra compared with IR7p-pre. No new peaks observed after modification of the sulfonic acid and lipoic acid groups. (B) The structure of molecule IR7p and IR7p-pre.

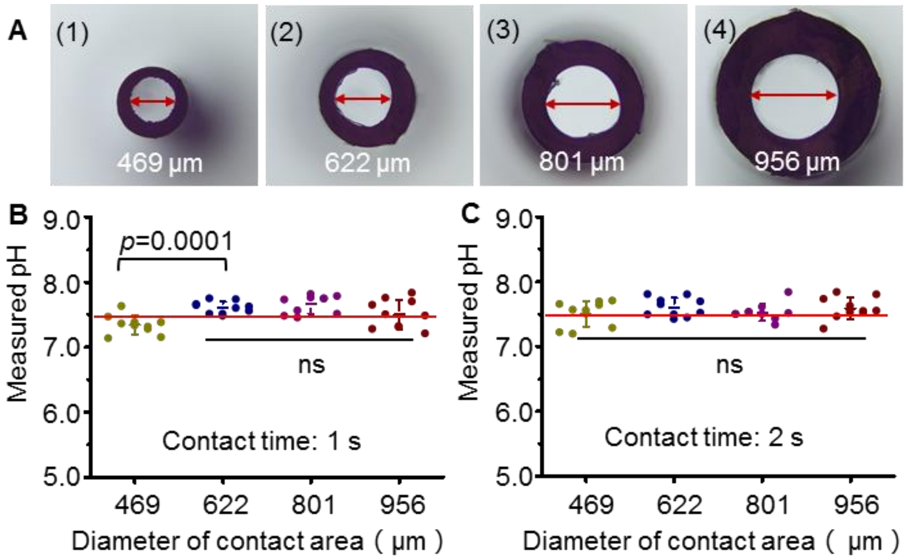


**Figure S6. The function of contact area of sampling pipette tip to the pH measurement accuracy.** The pH values measured from pipette tips with different contact areas. (A) Microscope images of the contacting areas of the pipette tips. The tissue contact times were 1 s (B) and 2 s (C) respectively. The data were analyzed by unpaired t test and one-way analysis of variance (ANOVA). ns: no significant difference.

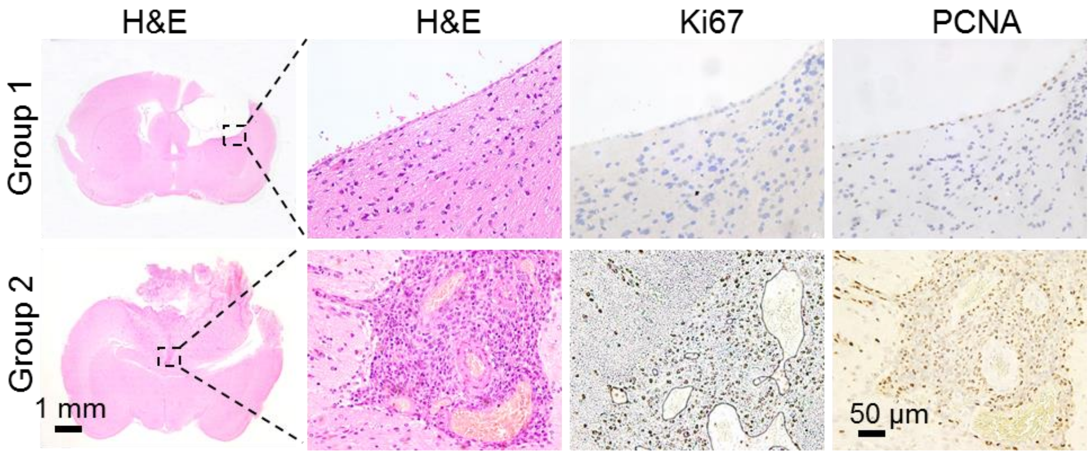


**Figure S7.** H&E and immunohistochemistry staining of rat model brain post the acidic margin guided surgery (Group 1, 180 days post resection) and preoperative gadolinium chelator guided surgery (Group 2, 25 days post resection) .

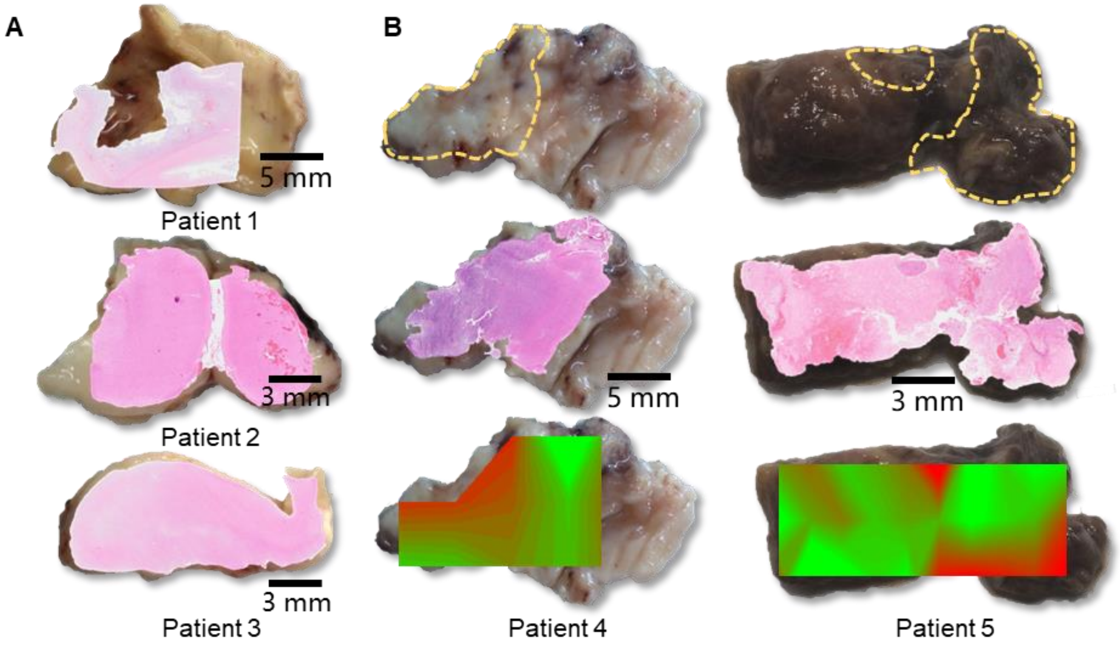


**Figure S8. Acidity correlated malignancy found in excised tissues from glioma patients.** (A) overlapped large visual field H&E staining of intra-operatively excised tumor slides from glioma patient 1–3. (B) White light photographic images, overlapped pH maps and overlapped H&E staining of intra-operatively excised tumor slides from glioma patient 4 and patient 5. Tumor margins were marked by yellow dotted lines.

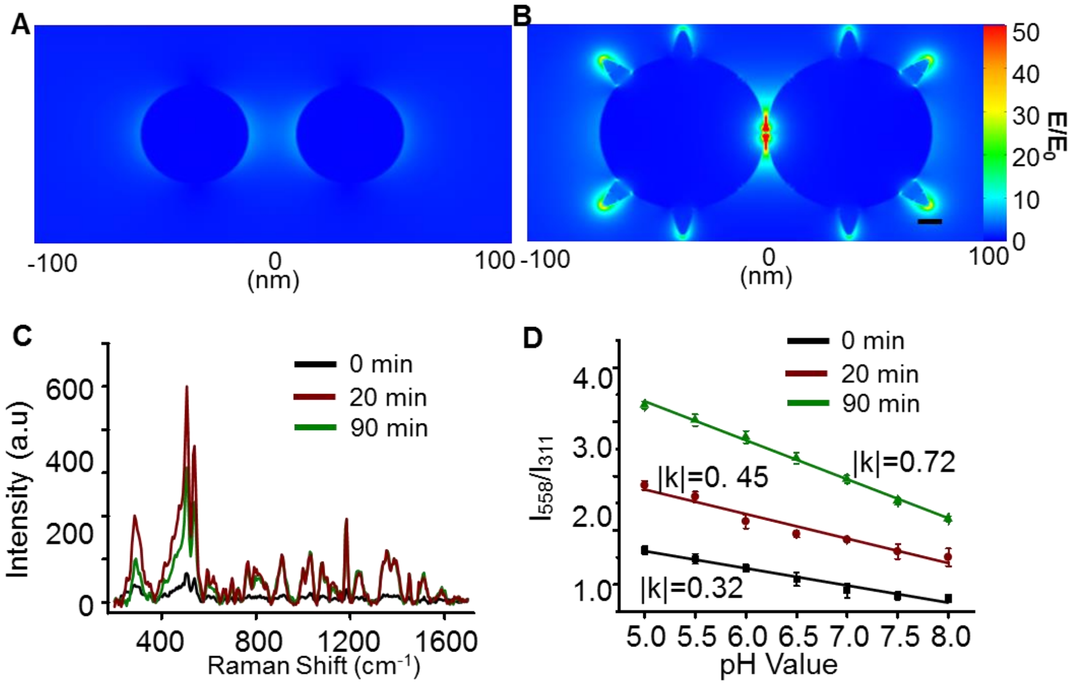


**Figure S9. The growth of gold nanoparticles increases the SERS intensity and the slope of fitting curves of SERS chip**. Finite difference time domain (FDTD) method simulated

electrical field intensity images at 785 nm for representative Au nanoparticle dimer growing for 0 min (A) and 90 min (B). (C) Raman spectra of SERS chip as a function of nanoparticle growth time. (D) Fitting curves of $I_{558}/I_{311}$ peak of SERS chips against pH value with different growth time.

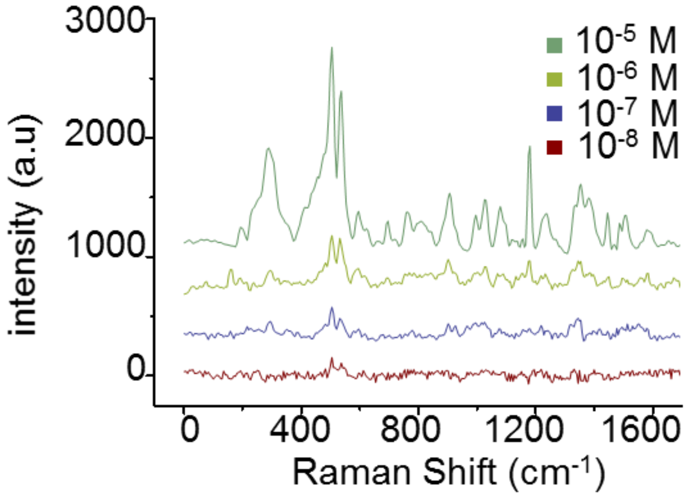


**Figure S10. SERS spectra of homemade SERS chips fabricated with IR7p with different concentrations.** Growth time: 90 min. Excitation wavelength: 785 nm, acquisition time: 1.0 s.

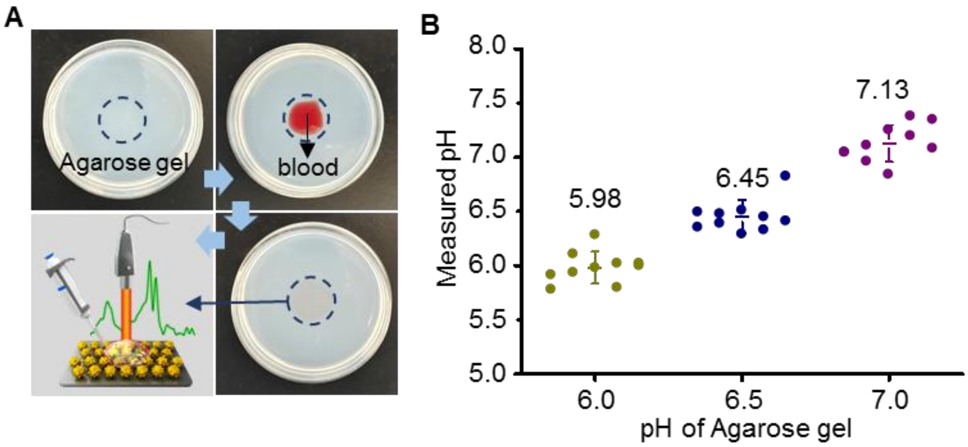


**Figure S11. Blood contamination barely changes the pH measurement accuracy in mimetic tissue.** (A) The diagram illustrating the experimental procedure. 10 μL fresh mouse blood was dropped on the agarose gel prepared with buffered solutions with pH 6.0, 6.5 and 7.0 respectively. The pH was measured before and 20 s after wiping the blood. (B) The pH values measured at the locations after wiping the blood were close to the original pH of agarose gel.

[1] A. F. Oskooi, D. Roundy, M. Ibanescu, P. Bermel, J. D. Joannopoulos, S. G. Johnson, *Computer Physics Communications* **2010**, *181*, 687.
[2] A. Farjadpour, D. Roundy, A. Rodriguez, M. Ibanescu, P. Bermel, J. D. Joannopoulos, S. G. Johnson, G. W. Burr, *Optics letters* **2006**, *31*, 2972.
[3] A. F. Oskooi, C. Kottke, S. G. Johnson, *Optics letters* **2009**, *34*, 2778.
[4] P. B. Johnson, R. W. Christy, *Physical Review B* **1972**, *6*, 4370.
[5] W. Duan, Q. Yue, Y. Liu, Y. Zhang, Q. Guo, C. Wang, S. Yin, D. Fan, W. Xu, J. Zhuang, J. Gong, X. Li, R. Huang, L. Chen, S. Aime, Z. Wang, J. Feng, Y. Mao, X. Zhang, C. Li, *Chemical Science* **2020**, *11*, 4397.
